# A case study on building capacity to improve clinical mentoring and maternal child health in rural Tanzania: the path to implementation

**DOI:** 10.1186/s12912-017-0252-0

**Published:** 2017-09-26

**Authors:** Melissa T. Ojemeni, Paulomi Niles, Salum Mfaume, Ntuli A. Kapologwe, Linda Deng, Renae Stafford, Marie Jose Voeten, Kokusiima Theonestina, Wendy Budin, Nok Chhun, Allison Squires

**Affiliations:** 1 Rory Meyers College of Nursing, New York University, 433 First Ave, 6th floor, New York, NY 10010 USA; 2 Shinyanga Regional Referral Hospital, Shinyanga, Tanzania; 3 Touch Foundation, Mwanza, Tanzania; 4 Sengerema Designated District Hospital, Sengerema, Tanzania; 5 Rutgers University, New Brunswick, NJ USA; 6 Yale University, New Haven, CT USA

**Keywords:** HRH, Women’s health, Clinical mentorship, Nursing education & workforce, Tanzania

## Abstract

**Background:**

Tanzania is a low income, East African country with a severe shortage of human resources for health or health workers. This shortage threatens any gains the country is making in improving maternal health outcomes. This paper describes a partnership between Touch Foundation and NYU Rory Meyers College of Nursing - Global, aimed at improving clinical mentorship and capacity among nurses and midwives at two rural hospitals in the Tanzanian Lake Zone Region. Clinical mentoring capacity building and supportive supervision of staff has been shown to be a facilitator of retaining nurses and would be possible to acquire and implement quickly, even in a context of low resources and limited technology.

**Methods:**

A case study approach structures this program implementation analysis. The NYU Meyers team conducted a 6-day needs assessment at the two selected hospitals. A SWOT analysis was performed to identify needs and potential areas for improvement. After the assessment, a weeklong training, tailored to each hospitals’ specific needs, was designed and facilitated by two NYU Meyers nursing and midwifery education specialists. The program was created to build on the clinical skills of expert nurse and midwife clinicians and suggested strategies for incorporating mentoring and preceptorship as a means to enhance clinical safety and promote professional communication, problem solving and crisis management.

**Results:**

Nineteen participants from both hospitals attended the training. Fourteen of 19 participants completed a post training, open ended questionnaire for a 74% response rate. Fifty-seven percent of participants were able to demonstrate and provide examples of the concepts of mentorship and supervision 4 and 11 months’ post training. Participants indicated that while confidence in skills was not lacking, barriers to quality care lay mostly in understaffing. Implementation also offered multiple insights into contextual factors affecting sustainable program implementation.

**Conclusions:**

Three recommendations from this training include: 1) A pre-program assessment should be conducted to ascertain contextual relevance to curriculum development; 2) flexibility and creativity in teaching methods are essential to engage students; and 3) access to participants a priori to program implementation may facilitate a more tailored approach and lead to greater participant engagement.

## Background

Tanzania is a low income, East African country with an estimated population of 45 million people which faces a severe human resources for health (HRH) or health workforce shortage [1]. The government has made inroads to increase HRH production over the past ten years by introducing new clinical cadres such as associate clinicians, reducing the educational training of nurses from four to three years and plans to triple the number of associate clinicians by 2020 [2, 3].

The HRH shortage has contributed to a dire state regarding maternal morbidity and mortality in the country. Tanzania is one of seven countries that account for 3–5% of global maternal deaths reported in 2010 [4]. Despite a 55% reduction in the maternal mortality ratio of 910 per 100,000 in 1990 to 410 per 100,000 in 2013 it remains a serious problem [4, 5]. In addition, Tanzania has an infant mortality rate of 36 deaths per 1000 live births and a lifetime risk of maternal death of 1 per 38 [4, 6–8]. Although Tanzania did not meet Millennium Development Goal 5: Improve Maternal Health, its government along with the public and private sector continue to heavily invest to increase access to maternal, newborn and child services nationally at all public health care facilities [5].

Health workers and particularly nurses, who comprise 27.8% of the Tanzanian health workforce, are important to reducing maternal mortality rates. Research has shown a correlation between too few HRH and adverse patient outcomes [9–11]. Research suggests one effective strategy for improving organizational level retention of health workers is strengthening clinical mentoring and supportive supervision of novice nurses and midwives [12, 13]. These “soft skills” are less dependent on technology and material resources and can be immediately applied to clinical and pedagogical practice.

In 2013, Vodafone Foundation launched Mobilizing Maternal Health (MMH) in Tanzania, an initiative to reduce maternal mortality. The Touch Foundation (a secular non-profit organization focused on improving Tanzanian health workforce capacity, health systems strengthening and maternal health outcomes) was selected by Vodafone as an implementing partner to reduce maternal morbidity and mortality. The Touch Foundation’s two partner hospitals in MMH target areas and the location sites for training implementation were: Sengerema Designated District Hospital (SDDH) and Shinyanga Regional Referral Hospital (SRRH). While the Touch Foundation was the primary implementing partner for MMH in Tanzania, they lacked the expertise in developing and implementing mentorship and supervision training. Touch partnered with the Global Division of Rory Meyers College of Nursing at New York University College (NYU Meyers) to provide their technical and educational expertise on HRH capacity building.

The purpose of this case study is to describe the development and implementation of a mentoring and supervision training program aimed at nurse-midwives, its monitoring and evaluation processes, and recommendations for future training in this area. Traditional mentoring can be defined as a relationship between a less experienced individual (the mentee) and a more experienced individual (the mentor) with the common goal of advancing the psychosocial and professional development of the mentee [14–16]. Mentoring programs can improve perceived support by health care workers, create opportunities for career advancement within an organization, improve retention and thus help an organization to maintain appropriate staffing levels that contribute to improved patient outcomes. [17–19] More recent theoretical explorations on mentoring point to the importance of viewing mentoring as mutually beneficial – providing opportunities for learning, development and growth for both mentors and mentees [20]. Psychosocial support functions are “those aspects of a relationship that enhance an individual's sense of competence, identity, and effectiveness in a professional role” ([15] p. 32). Integrating these skills into the repertoire of experienced clinicians has the potential to improve recruitment and retention of less experienced personnel by contributing to a more positive and collaborative work environment.

## Methods

We use a case study approach to organize our description of the program implementation and evaluation processes. Case studies are an effective method for organizing an analysis where multiple sources of data are needed to capture a phenomenon, but where the context of data collection is less structured and cannot conform to the conditions of other more structured research approaches [21, 22]. In this section, we describe the program planning efforts to establish how implementation was conducted and the analytic approach used to evaluate the program. There was no theoretical framework used to guide this study since case studies do not require them [23].

### Needs assessment

#### Pre departure preparation

Prior to performing the six day in country needs assessment the principal investigator and remaining members of the NYU Meyers team were in constant communication with Touch Foundation via conference calls and email to organize logistics and expertise on teaching needs that could be anticipated at each hospital. The NYU Meyers team was comprised of four women with extensive international health experience, two PhD prepared nurse scientists with over 20 years’ combined experience in health services research and maternal child health and 2 Master’s prepared nursing PhD students with over 15 years’ experience in midwifery and bedside clinical care experience.

#### IRB approval

IRB and/or ethical approval was deemed exempt by the home institution as it was a descriptive case study and evaluation of an educational training program and did not involve the collection of sensitive information.

#### In country needs assessment

A strength, weakness, opportunities and threats (SWOT) analysis (Table 1) technique was used to provide a rapid assessment of each hospital and to help structure the needs assessment approach. The SWOT analysis’s intent was to provide a snapshot of short, medium and long term insights on the current situation for both the in-country partners and hospital administration based on what the assessors observed and obtained from the need assessment. It was strictly context for all parties involved with program implementation. The actual scope of the clinical mentorship and supervision program, although small, served as one component to Touch’s overarching goal to reducing maternal mortality in Tanzania. Thus, the SWOT analysis provided in this manuscript should be interpreted with caution with regard to the program described in this paper.

**Table 1:** SWOT analysis from needs assessment of Shinyanga Regional Referral Hospital

Strengths	Weakness
*Organized, management team at both the hospital and district level* • Planned establishment of a birthing home to accommodate women living very far who want to deliver in the hospital • Daily interprofessional ward rounds currently take place • Hospital orientation in place for new nurse hires; 6-month process that functions like a new graduate internship program • 3-year contract required by all new staff	• High demand for resources-human, structural and supplies • Nationally, has some of the worst statistics related to maternal and infant mortality but trending improvements • Partograph competency low • Reinforcement of practical skills in neo-natal resuscitation; managing a patient with complex care issues • Quality of care challenges due to HR staffing, recruitment, and retention issues Change in nursing education (reduced time) found to affect quality of care provided by graduates; poor coordination with local facility to address needs
Opportunities	Threats
*Patient Education to Improve Outcomes* • Provide educational sessions in key problem areas (e.g. nutrition, paternal care of the pregnant woman) for patients who are awaiting care in the wards or clinics *Long term operational improvement opportunities* ○ Creating a conducive environment for students to learn since they are vital to assisting with delivery of care on units ○ Implement a clinical ladder program to improve internal career development opportunities and improve retention • Establish a 3 month, short term volunteer program for internationally educated nurses & other healthcare providers	• Ability to measure long term impact of this training; whether this training will be able to make a difference given the severity of the nursing shortage and the volume of deliveries per month in the hospital • Lack of digitized data for analysis to examine the impact of various organizational interventions or staffing changes on patient outcomes in the facility • High staff turnover

The needs assessment was conducted in July 2014 over a six-day period. Once in country, the two assessors met formally with Touch Foundation personnel and provided a layout of the assessment process for the upcoming week. While the initial project sought to implement basic emergency obstetric and newborn care (BEmONC) and comprehensive emergency obstetric and newborn care (CEmONC) training, those plans changed due to a new policy mandated from the Ministry of Health and Social Welfare (MOHSW). The new policy specified that MOHSW is the only body that can deliver training for emergency obstetric and newborn services in accordance with the national curriculum. The NYU Meyers team was made aware of the change upon arrival for the needs assessment. Based on the findings from the needs assessment and the critical staffing shortage present at each facility, the team changed the training focus on providing and improving clinical mentorship capacity among the nurses and midwives. The final needs assessment drew from evidence-based approaches to effective maternal child health (MCH) service delivery and organizational evaluations sensitive to the dynamics of nursing personnel working in a low resource, interprofessional clinical setting.

To gain an understanding of what was taking place at SDDH and SRRH specific to MCH, the NYU Meyers assessors engaged in conversations with stakeholders which included nursing, midwifery and hospital management, staff, medical directors and district health officials. Informal interviews were held with four key informants at SDDH and eight at SRRH. A semi-structured interview guide was used to elucidate information about the concerns, long term plans and needs of each facility. Team members took notes during the interviews and did not record them. Hospital and unit tours allowed the assessors to observe the realities of nursing and midwifery practice in an effort to generate potential recommendations to help deliver better patient care. Data notebooks with labor and delivery statistics were reviewed for the types of data collected by each hospital. Assessment sheets with delivery statistics were completed within one week by hospital personnel and emailed back to the NYU Meyers team.

### Curriculum development

After conducting the needs assessment, the NYU Meyers team created a curriculum that encapsulated the needs of all stakeholders (Touch, hospital administration and nursing staff). The primary goal of the program was to help nurses and midwives to refresh and improve their clinical competence while incorporating principles of mentoring and clinical supervision throughout the training. Importantly, the nursing and midwifery staff determined the priorities for the clinical refresher portion of the program. Some of the clinical content that staff wanted reviewed were complications during labor and delivery such as neo-natal resuscitation and postpartum hemorrhage. The Touch Foundation was provided with drafts of the curriculum for review and feedback prior to implementation.

The week-long training was designed and taught by two NYU Meyers nursing and midwifery education specialists. Clinical case studies served as the curriculum framework to discuss how mentorship and clinical teaching could be integrated into an overburdened environment to enhance professionalism and support evidenced based practice. Enhancing mentorship and clinical supervision was a central theme to all learning objectives and exercises. Using methods beyond the traditional didactic method was also key to the week-long training. Methods such as teach back, small group work, demonstrations, role plays, and low technology simulation were actively utilized. While common place in Western settings, these methods were novel to many of the Tanzanian participants. Although the NYU Meyers team tailored the curriculum to meet the needs voiced by our Tanzanian counterparts, we recognized that we were coming from a Westernized perspective and wanted to try and limit the potential for any bias in content delivery and adapt to local learning styles.

### Program implementation

#### Setting

Two sites in rural, northern Tanzania participated in the program. *Sengerema Designated District Hospital* ***.*** Established in 1959, SDDH is a 318-bed Catholic hospital that offers public services to over 700,000 people in Sengerema district. SDDH joined Touch Foundation’s *Treat & Train* Network in 2013 and was the first hospital to host Touch Foundation’s external clinical rotations for healthcare students. SDDH attends to over 10,000 births per year and, in 2014, was the first hospital to pilot and implement Vodafone’s MMH program. The training at SDDH occurred in August 2014.

*Shinyanga Regional Referral Hospital* served as the second site*.* SRRH was built in the 1940s and is a government owned 300-bed hospital serving a catchment area of over 1.5 million people. Shinyanga has been a part of Touch’s *Treat & Train* Network since 2014 and is now hosting external clinical rotations for healthcare students as well as implementing the MMH program for the Shinyanga District Council. SRRH attends to almost 8000 births per year. The training at SRRH occurred in January 2015.

#### Participants

After the needs assessment and discussion with Touch it was decided that the nurses and midwives serving in hospital administration and leadership roles, who also still practiced on the wards, were best suited to select training participants. Participants were purposively recruited for the program.

#### Program implementation

The overall purpose of the training program was to prepare and engage nurses and midwives to nurture and support novice providers and students in delivering optimal care to the women and babies they serve in low resource, high intensity settings. The objectives for the training participants included: 1) defining mentoring and developing an awareness of their preexisting mentoring style; 2) developing an action plan, defining objectives and goals to enhance learning and skills acquisition for novice clinical staff; 3) utilizing essential obstetrical assessment and decision making tools in managing care; and 4) incorporating various teaching techniques to support engaged learning. Core concepts such as communication, interdisciplinary teamwork, trust building and professional leadership in relation to professional development and workforce strengthening were also reinforced. Exploring underlying care values and enhancing respectful maternity care were also subthemes that were intentionally integrated into lesson plans throughout the weeklong training.

Each training, one at each site, took place over five days and were four hours per day. In addition, there was up to one hour per day allocated for clinical review of the course content. Table 2 provides a sample curriculum outline to provide context of what was taught during the training. Supplementary materials – such as theme appropriate articles – were introduced and well received by the group. Teaching and mentoring exemplars in the program emphasized how to coach and teach less experienced staff core concepts around improving patient safety for obstetric and neonatal care, including urgent care situations.

**Table 2:** Sample Program Curriculum

DAY 1	DAY 2	DAY 3	DAY 4	DAY 5
Opening Welcome & Introductions Course Overview Presentation & Discussion Intro to Mentoring	Agenda & Opening Activity Review and Clarify Presentation & Discussion Effective Communication Using SBAR	Agenda & Opening Activity Review and Clarify Presentation & Discussion Empowering Others through Effective Supervision	Agenda & Opening Activity Review & Clarify Presentation & Discussion Skills & Drills: Neonatal Resuscitation	Agenda & Opening Activity Review & Clarify Presentation & Discussion Having a Plan: Creating Action Plans for Teaching and Mentoring Creating a Culture of Caring
*BREAK*	*BREAK*	*BREAK*	*BREAK*	*BREAK*
Presentation & Discussion Mentoring Process: review of Basic Principles Learner Self-Assessment:	Presentation & Discussion Building Relationships	Presentation & Discussion Using Case Studies: Teaching through Clinical Stories	Presentation& Discussion NRP Review Hands on Teaching Methods Drills/Simulations for Enhanced Learning	Closing Evaluation Certificate Vows
Readings: 1. State of the World’s Midwifery 2014: Country Brief Tanzania 2. Improving Partograph use in Uganda through coaching and mentoring: *Engender Health, 2013*.	Readings: 1. Coleman, D (1998) What makes a leader? *Harvard Business Review, 1998*. 2. The Mentoring Manifesto, David Cohen	Readings: (Optional) 1. McConnell, C. Delegation versus empowerment: What, how and is there a difference. *Health Care Survey, 1995*.		

Although the language of instruction in Tanzania is English and each facility assured the NYU Meyers team that the participants could engage in English, all pre-assessment and post questionnaire documents were in both English and Kiswahili. Clinician-champions, exemplar participants, identified by NYU Meyers educators were proficient in both spoken and written English and Kiswahili were also on hand and able to provide translation during classroom instruction. This step helped ensure there would be minimal issues related to translation.

## Results

This section will provide results of both the participant completed assements and the programs' evaluation. In total, 19 nurses and midwives, nine at SDDH and ten at SRRH, participated and completed the trainings. Seventeen were women and two were men. There was a 100% attendance rate for all participants. Clinician-champions, exemplar participants who could help ensure the sustainability of the program, were identified at both sites by training instructors.

Prior to the program’s start, participants had completed a self-assessment to measure their clinical competence and barriers to practice in their hospital setting. Overall, participants from SDDH and SRRH self-reported that they were very confident in their clinical skills and their ability to provide competent care to both pregnant women and their newborn babies. Lack of staffing and resources were the two main barriers limiting participants from practicing within their full scope. Poor provider to patient relationships and inadequate staffing, as identified by the participants, emerged as major barriers affecting nurse to patient interactions that involved history taking, encouraging mothers to breastfeed or providing patient updates to family members.

Initial, post-training evaluations, completed at the end of each week-long training on site, were positive with participant comments on new knowledge acquired in mentoring, clinical case management, and newborn resuscitation. The initial training evaluation provided anecdotal results from participants who emphasized that they particularly enjoyed “the way the teachers taught us demonstration of newborn resuscitation” and “mentoring.” Participants did emphasize that they wanted an “increase period for training.” The participants at both sites believed the educational climate was conducive to learning, the instructors were prepared and the students felt they were in a comfortable environment to engage and ask questions.

After each training, monthly and bimonthly emails were sent to follow-up with participants, elicit their feedback and ascertain their ability to incorporate mentoring principles into their practice. These attempts proved unsuccessful. The team concluded that technological difficulties (access to reliable internet) and a lack of in-country presence post-training implementation were primary factors affecting poor follow-up. A 6-month in-person follow-up with participants at SDDH was scheduled for January 2015, with clinical champions assisting to coordinate the follow-up. Participants were notified in advance, were aware and expressed interest in attending; however, no participants were able to be located or available during the follow-up visit.

Having learned from the unsuccessful post-program evaluation attempts from the initial 6-month follow-up, Touch Foundation played a more integral role in assisting with follow-up by distributing and collecting the post questionnaire evaluation forms from providers at each hospital. It was administered in-person by Touch Foundation 11 months after the SDDH training and 4 months after the SRRH training. The post training questionnaire consisted of 11 open ended questions to allow participants to demonstrate: 1) knowledge acquisition and retention about mentoring and supervision after the training; 2) how they have been able to incorporate the training into their daily practice; and 3) recommendations they would make for future trainings.

Fourteen out of nineteen participants returned the questionnaires for a 74% response rate. Amongst the respondents, 57% of participants were able to demonstrate proficiency on the concepts of mentorship and supervision, provide examples of action plans on how they could address challenges on their wards and mentor students and novice nurses. In the post questionnaire, participants also highlighted the workforce shortages, lack of professional development for themselves and their co-workers at each site as impediments to their professional practice. Interestingly, 7 out of 14 participants also mentioned language as being a barrier but they did not specify if it was a patient-to-provider or provider-to-provider barrier in terms of language, a finding that needs further exploration.

## Discussion

The results from the program and its implementation offer important insights and reminders for organizations seeking to implement similar programs in Tanzania. The lessons may also be applicable to other low income country contexts, but would require further testing as these findings are largely qualitative and not generalizable.

Nonetheless, the program was well received by the staff, hospital administration and local government officials at both sites. The staff particularly were enthusiastic, willing to learn, engaged, professional and welcoming. The facilities were very supportive of the program, accommodated the training by providing space and tried to ensure that supplies were provided if needed. This program highlighted the importance of expanding professional development beyond tactile skills and was innovative in that it provided participants with hands on learning experiences, bringing both the tactile and soft skills through this mentorship program. It also provided an opportunity to diversify and expose participants to various teaching styles such as case studies, hands on demonstration and the use of low tech simulation.

### Program implementation barriers

Despite the positive feedback from participants, there were some implementation barriers identified. For the program instructors, the cultural expectation of payment/compensation from the providers to attend the training proved to be difficult to handle and at times distracting from the lessons taking place. This expectation by staff is not uncommon as it has been found in other studies [24, 25]. In addition, the poor turnout of participants for evaluative follow-ups is an outcome that has also been consistent in other studies [26, 27]. Coupled with no incentives to participate and staffing shortages, these issues may have also factored into participants decisions not to attend.

#### Areas for improvement

For future similar programs such as this, a pre and posttest should be conducted to assess knowledge gains. Although application in the wards would not be able to be assessed through a pre and posttest there would at least be the opportunity to monitor progress at baseline. In addition, daily feedback would have been sought from participants to re-evaluate and tailor the daily program curriculum instead of an evaluation at the end of the five day course. By the 2nd day of the program, champions would have been identified by instructors and they would have one additional day of mentorship individually with the trainers with continued mentorship one day a month via distance learning with program instructors.

After the formal training ends, these identified champions could provide reinforcement trainings every three months on particular modules that need refreshment. These champions could assist with follow-up surveys at each site and be a liaison with the program instructors to provide follow-up with their colleagues about what is working and not working on the wards. The champion mechanism would require buy in from in country partners and each facility would provide this individual with dedicated time away from the bedside and focus on education, follow-up and their continued professional development. At six months, the Champions can administer a second posttest to check on knowledge attainment at that point. With these types of partnerships, in country partners could also serve as a support system to the champions and continue to provide professional development opportunities that they would be expected to bring back to their facilities. In addition, or as an alternative to the pre and posttest evaluation mechanism, there should be consideration for doing a pre and post program implementation focus groups with providers at 3 and 6 months intervals. Integrating this type of follow-up would shed light on the sustainability of the program and potential for long-term effectiveness.

While the program was able to demonstrate modest gains in knowledge attainment, a number of limitations with the evaluation data should be noted. NYU Meyers’ limited in country presence after each training was a major hindrance to follow-up with the participants. The sample size was too small for meaningful statistical comparisons and generalizability, but this is common with small scale training programs like this one. In addition, poor internet and electrical power infrastructure contributed to infrequent communication with participants thereby hampering a thorough follow-up to compare pre and post program objectives and goals. More ongoing collaboration with in country stakeholders would have benefited curriculum development but infrastructure limitations hampered that possibility. In addition, the trainings’ dramatic shift from a BEmONC and CEmONC training to a mentoring and supervision training required a rapid restricting and remodeling of expectations, objectives and goals for both Touch Foundation and NYU Meyers.

Despite the limitations, the program’s successes were sufficient that the Touch Foundation is exploring expansion into local health centers around each hospital since the original design was structured to be replicable across sites. To facilitate this goal, the NYU Meyers team created a fully decentralized curriculum of shorter modules from the training for expansion to various settings beyond the two original sites. Themes, skills and knowledge covered relate to strengthening the capacity of health care providers to effectively mentor new students and clinicians. On a national level, current efforts between the Tanzanian Ministry of Health, Community Development, Gender, Elderly and Children and JHPIEGO, an international non-profit, are also aiming to increase clinical mentoring on a national scale.

The common threat to any sustainable HRH capacity building program at both sites is the staffing, recruitment and retention of nursing and midwifery personnel. In resource constrained environments, investing time and energy in newer clinicians can offer much needed support and safety to the patients they care for [28, 29]. Building the supervisory and mentoring capacity of expert clinicians can have a ready and measurable impact on the functionality, efficiency and quality of new HRHs [30–32].

One change that might improve sustainability is that in-country partners or partnering institutions should consider having a line item to include compensation or other perks for providers who participate in program such as this one. If a financial incentive could not be provided, continuing education, cell phone air time, or usb flash drive could be used as incentives. If incentives cannot or will not be provided, participants should be advised well in advance.

Finally, partnerships that involve one partner outside of the country where a program will be implemented should have transparency and open communication. For new partnerships, conversations should be had around logistical support for partners while in country to help troubleshoot issues (lodging, transport, copying documents, etc., translations) or provide contacts that could be of assistance. In addition, a liaison or guide at each sight to help trainers or partners get acclimated for at least the first 72 h would be highly desirable. Translators can also be beneficial if the primary language is not spoken by team members from outside of the country. For future programs that will have a needs assessment and program implementation component it is advised that at least one program instructor also conduct the needs assessment as a content expert. If identifying a champion at each site is not viable working with the hospitals to create a full time nurse educator position would be greatly beneficial to continue professional development on site and reinforcing curriculum content.

## Conclusions

Three recommendations have emerged from the program’s implementation: 1) curriculum development in low resources settings should involve an assessment of the clinical and practical needs of the participants and include their input into the curriculum design and content; 2) flexibility and creativity in teaching methods are essential to engage students; and finally, 3) access to participants a priori to program implementation may facilitate a more tailored approach and lead to greater participant engagement. It is important to note that these recommendations should be taken with caution as country context and partnership goals may vary depending on program objectives. It is important for both partners particularly those vested in the long term to have an in country presence to assist with follow-up communication and responding quickly if things change in country.

Health workers’ ability to educate future generations of Tanzanian health care providers must be enhanced and sustained to maximize gains for the women they serve. The shortage of human resources threatens the effectiveness and sustainability of efforts to build capacity among staff. Offering curriculum development and implementation support– through university and non-profit partnerships – may offer a key strategy to filling a need to support nurses and midwives who work under resource-strained conditions.

## Abbreviations

BEmONC: Basic Emergency Obstetric and Newborn Care
CEmONC: Comprehensive Emergency Obstetric and Newborn Care
HRH: Human Resources for Health
MCH: Maternal Child Health
MMH: Mobilizing Maternal Health
MOHSW: Ministry of Health and Social Welfare
NYU MEYERS: New York University of Nursing
SDDH: Sengerema Designated District Hospital
SRRH: Shinyanga Regional Referral Hospital
SWOT: Strengths, Weakness, Opportunities and Threats
